# Comparative Analysis of Codon Usage Bias in Six *Eimeria* Genomes

**DOI:** 10.3390/ijms25158398

**Published:** 2024-08-01

**Authors:** Yu Zhao, Shicheng Zhang

**Affiliations:** College of Life Science and Technology, Gansu Agricultural University, Lanzhou 730070, China; kk021116@outlook.com

**Keywords:** *Eimeria*, genome, codon usage bias, natural selection, mutation pressure

## Abstract

The codon usage bias (CUB) of genes encoded by different species’ genomes varies greatly. The analysis of codon usage patterns enriches our comprehension of genetic and evolutionary characteristics across diverse species. In this study, we performed a genome-wide analysis of CUB and its influencing factors in six sequenced *Eimeria* species that cause coccidiosis in poultry: *Eimeria acervulina*, *Eimeria necatrix*, *Eimeria brunetti*, *Eimeria tenella*, *Eimeria praecox*, and *Eimeria maxima*. The GC content of protein-coding genes varies between 52.67% and 58.24% among the six *Eimeria* species. The distribution trend of GC content at different codon positions follows GC1 > GC3 > GC2. Most high-frequency codons tend to end with C/G, except in *E. maxima*. Additionally, there is a positive correlation between GC3 content and GC3s/C3s, but a significantly negative correlation with A3s. Analysis of the ENC-Plot, neutrality plot, and PR2-bias plot suggests that selection pressure has a stronger influence than mutational pressure on CUB in the six *Eimeria* genomes. Finally, we identified from 11 to 15 optimal codons, with GCA, CAG, and AGC being the most commonly used optimal codons across these species. This study offers a thorough exploration of the relationships between CUB and selection pressures within the protein-coding genes of *Eimeria* species. Genetic evolution in these species appears to be influenced by mutations and selection pressures. Additionally, the findings shed light on unique characteristics and evolutionary traits specific to the six *Eimeria* species.

## 1. Introduction

In the amino acid composition of organisms, individual amino acids may correspond to multiple codons, a phenomenon known as codon degeneracy. Codons encoding the same amino acid are termed synonymous codons. Despite codon degeneracy allowing for multiple codons to encode a single amino acid, various organisms exhibit distinct preferences for specific synonymous codons. The codon serves as the fundamental unit of information for mRNA translation, with 62 codons encoding 20 distinct amino acids [1]. However, across various genes or genomes, the selection of synonymous codons exhibits a non-random pattern, different organisms exhibit preferences for specific codons during amino acid encoding, reflecting a phenomenon known as codon usage bias (CUB) [2]. Although synonymous mutations were traditionally viewed as “silent” mutations due to their lack of impact on protein sequences, research suggests that codon selection during evolution is not entirely neutral [3,4]. For specific species, certain synonymous codons, termed optimised codons, are favoured, while others are used less frequently. Furthermore, codon usage patterns can impact various biological processes such as mRNA synthesis, the rate of translation elongation, protein folding, and other subsequent cellular functions [5,6,7]. Specific synonymous substitutions have notable fitness and phenotypic effects across various organisms, including vertebrates and invertebrates [8].

It is widely recognised that CUB is mainly influenced by mutation pressure, natural selection, and random genetic drift [9,10]. This preference is closely linked to GC content, gene expression level, gene length, tRNA abundance, protein structure, and RNA stability [11,12,13,14]. For instance, highly expressed genes tend to favour codons that match abundant tRNAs, resulting in CUB. The development of CUB is influenced by the interplay between translational selection and mutation pressure [15,16]. Analysing codon usage patterns can offer insights into the evolutionary and adaptive processes of different species, as codon usage may vary among species or even within the same species due to different evolutionary pressures [17]. Additionally, investigating the CUB of pathogens can offer valuable insights into the regulation of pathogenic gene expression, thus contributing to the advancement of more effective vaccine strategies.

Chicken coccidiosis is a prevalent and severe parasitic disease among poultry [18]. It manifests as an acute epidemic protozoal infection caused by one or more species of coccidia [19]. This disease poses a significant threat to young chicks and is particularly prevalent among chickens aged 20–45 days [20]. Its incidence is highest during seasons characterised by temperatures of 25–30 °C and heavy rainfall [21]. The occurrence rate of coccidiosis can reach up to approximately 75%, with mortality rates ranging from 20 to 50% [22]. Affected chicks experience stunted growth and slow weight gain upon recovery. While adult chickens typically remain asymptomatic, carriers may exhibit reduced weight gain and egg production, thereby serving as important vectors for coccidiosis [23]. Chicken coccidiosis inflicts substantial economic losses annually on the global poultry industry [24,25]. Chicken coccidiosis is an intestinal parasitic infection caused by one or more species of coccidia belonging to the phylum Apicomplexa, class Sporozoa, order Eucoccidiorida, family Eimeriidae, and genus *Eimeria* [26]. Globally, seven species of chicken coccidia have been identified, including *Eimeria acervulina*, *Eimeria necatrix*, *Eimeria brunetti*, *Eimeria tenella*, *Eimeria praecox*, *Eimeria maxima*, and *Eimeria mitis* [27]. These various species exhibit differing patterns of parasitism and pathogenicity. Notably, *E. necatrix* and *E. tenella* are among the most pathogenic species, with *E. necatrix* predominantly parasitising the mid-portion of the small intestine and *E. tenella* inhabiting the ceca [28].

At present, there are six complete genomes of the genus *Eimeria* that have been sequenced and reported. This study aims to analyse the genome-wide codon preferences of six *Eimeria*-encoded proteins using programming languages and bioinformatics tools. By comprehensively comparing codon usage patterns, this study seeks to enhance heterologous gene expression, improve resistance to coccidiosis, and facilitate the development of vaccines against parasitic diseases. Additionally, this research aims to lay the groundwork for functional genomics and phylogenetic studies in *Eimeria*, contributing to our understanding of gene origin, protein expression, and gene evolution processes.

## 2. Results

### 2.1. Analysis of Nucleotide Composition and Codon Usage in Eimeria

The GC content of the protein-coding genes among the genome of six *Eimeria* species ranged from 52.67% to 58.24%, with *E. maxima* having the lowest value and *E. necatrix* having the highest. The coding sequences (CDSs) in the six *Eimeria* species exhibited a higher abundance of G and C nucleotides compared to A and T nucleotides. The average GC1 (GC content at the first position of codons) content exceeds that of both GC3 (GC content at the third position of codons) and GC2 (GC content at the second position of codons) in every species, with the distribution trend as GC1 > GC3 > GC2 (Table 1). The GC1 contents of six *Eimeria* species ranged from 61.99% to 66.09%, among which *E. maxima* had the lowest value and *E. brunetti* had the highest value. The GC3 contents of six *Eimeria* species ranged from 48.71% to 59.75%, among which *E. maxima* had the lowest value and *E. tenella* had the highest value. In terms of GC2, the GC2 contents ranged from 45.84% to 50.40% among six *Eimeria* species, while *E. praecox* had the lowest value and *E. necatrix* had the highest value. Comparable trends in nucleotide composition were noted in the third positions of synonymous codons, the GC3s (GC content at the third position of synonymous codons) content ranged from 47.43 (*E. maxima*) to 58.80 (*E. necatrix*), with the exception of *E. maxima*, the values for the other five *Eimeria* species exceeded 50%.

**Figure 1:**
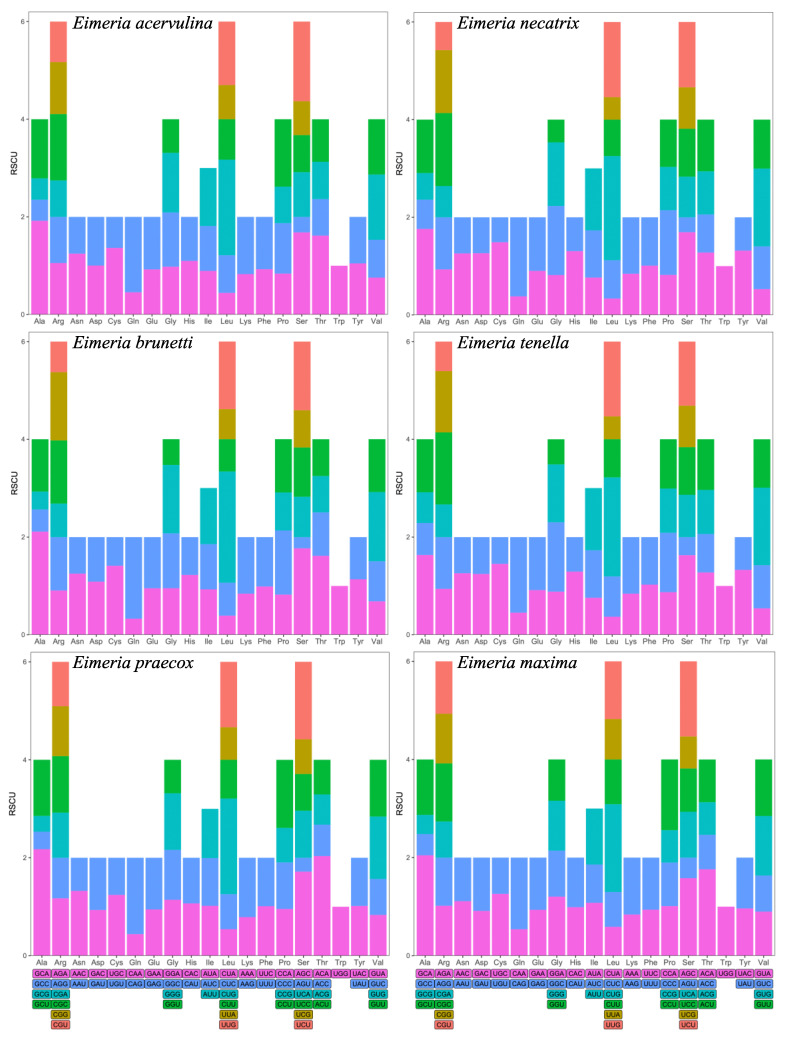
An examination of RSCU was conducted on protein-coding genes derived from six *Eimeria* species, specifically, *E. acervulina*; *E. necatrix*; *E. brunetti*; *E. tenella*; *E. praecox* and *E. maxima*.

The overall RSCU (relative synonymous codon usage) value of the six *Eimeria* genome was calculated (Figure 1). There are 26 codons with RSCU values greater than 1 in *E. acervuline*, *E. necatrix*, and *E. tenella*. Both *E. brunetti* and *E. praecox* contain 27 codons with RSCU values greater than 1. In addition, 28 codons with RSCU values greater than 1 were found in *E. maxima*. Among these high-frequency codons of *Eimeria*, most codons end with C/G. However, in *E. maxima*, codons tend to end with A/T more than C/G in high-frequency codons. This is related to the fact that only GC3 in *E. maxima* is below 50% among six *Eimeria* species. At the same time, the RSCU value of 31 codons is less than 1 in *E. maxima*, and the RSCU value of 32 codons is less than 1 in *E. brunetti* and *E. praecox*. *E. acervuline*, *E. necatrix*, and *E. tenella* have 33 low-frequency codons. Among these low-frequency codons, except in *E. maxima*, most of them tend to end with A/T. 

### 2.2. Assessing the Correlation between Codon Usage Metrics

**Figure 2:**
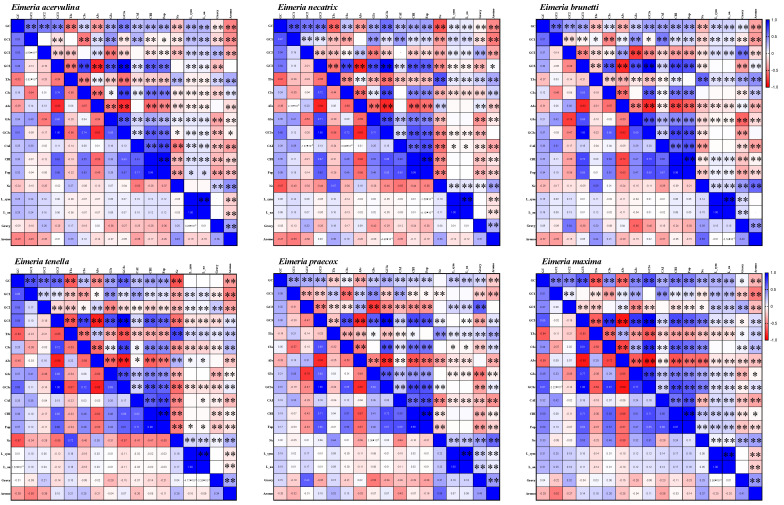
Correlation analysis among different indices across six *Eimeria* species. Dark blue indicates a positive correlation, while dark red indicates a negative correlation. A higher value indicates a more significant correlation. Asterisks (*) denote statistically significant correlation alterations between the two indicators at a significance level of *p* < 0.05, and double asterisks (**) indicate significant correlations at the *p* < 0.001 level. The six *Eimeria* species, listed from left to right and from top to bottom, include *E. acervulina*; *E. necatrix*; *E. brunetti*; *E. tenella*; *E. praecox* and *E. maxima*. T3s, C3s, A3s, GC3s: compositions of third synonymous codons. CAI: codon adaptation index. CBI: codon bias index. Fop: frequency of optimal codons. Nc: effective number of codons. L_sym: number of synonymous codons. L_aa: length amino acids. Gravy: grand average of hydropathicity. Aromo: aromaticity.

We observed a significant positive correlation between GC3 content and GC3s across six *Eimeria* species (*p* < 0.001). Additionally, there was a significant positive correlation between GC3 or GC3s and CBI (codon bias index) across these species. Furthermore, we noted a notable negative correlation between GC3 and A3s, but a significant positive correlation between GC3 and C3s in the same set of *Eimeria* species (*p* < 0.001). Moreover, CBI exhibited a significant positive correlation with FOP (frequency of optimal codons) across all *Eimeria* species (*p* < 0.001). Additionally, the L_sym (number of synonymous codons) index showed a significant positive correlation with the L_aa (length amino acids) index across all six *Eimeria* species (*p* < 0.001). The results indicate that the nucleotide composition can impact the CUB of genes in *Eimeria* species (Figure 2).

### 2.3. ENC-Plot Analysis

The average ENC values of the six *Eimeria* species ranged from 47.37 ± 8.70 to 51.93 ± 5.65, with *E. praecox* having the lowest value and *E. acervulina* having the highest. The average ENC values were 47.67 ± 7.89 for *E. brunetti*, 49.30 ± 7.28 for *E. necatrix*, 50.25 ± 6.97 for *E. tenella*, and 51.17 ± 6.97 for *E. maxima*, suggesting a general random codon usage pattern across the *Eimeria* genomes (Table 1). Among six *Eimeria* species, the ENC value of from 1.22% to 7.32% of the genes was less than 35, with *E. brunetti* having 7.32% of genes, and *E. acervulina* having 1.22% of genes, indicating that these genes within each species have a strong codon bias. 

**Figure 3:**
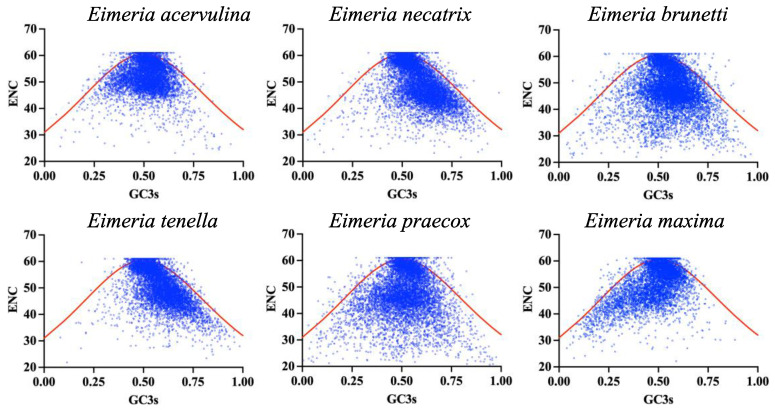
The ENC-plot analysis was conducted on protein-coding genes across six *Eimeria* species. The red solid line in the plot represents the expected curve under the assumption that codon usage bias is solely influenced by mutation pressure. The species analysed include *E. acervulina*; *E. necatrix*; *E. brunetti*; *E. tenella*; *E. praecox* and *E. maxima*.

To assess the relationship between synonymous codon usage patterns and Enc across all genes within each *Eimeria* genome, the ENC-plot was constructed. The results showed that most genes in each species were located far below the expected ENC-plot curve, and only a small number of genes fell onto the expectation curve (Figure 3). This analysis revealed that the main factor affecting the CUB was selection pressure in six *Eimeria* species, at the same time, only a small number of coding genes are solely due to mutational pressure that leads to changes in codon usage.

We also calculated each species’ ENC frequency distribution in six *Eimeria* to test the discrepancy between observed ENC (ENC_obs_) and expected ENC (ENC_exp_) values. This analysis revealed that 70.51~82.20% of genes are distributed outside from −0.05 to 0.05, *E. brunetti* and *E. praecox* genomes had ratios greater than 80%, while the remaining four genomes had ratio values between 70.51% and 76.59%. These data show that the main factor affecting the codon usage in most protein-coding genes of six *Eimeria* species is natural selection pressure, and some genes are also affected by mutational bias, which further suggests that the formation of codon bias within these genomes were largely responsible for GC3s.

### 2.4. PR2-Plot Analysis

The PR2-plot analysis was conducted to assess biases in the third codon position within four codon degenerate amino acids among protein-coding genes across six *Eimeria* species. According to Chargaff’s second parity rule (PR2), the quantities of A = T and C = G in a DNA strand are equivalent [29]. Each data point on the plot represents a gene, with the plot segmented into four quadrants. The centre of the plot, where both coordinates are 0.5, denotes the equilibrium point where A = T and G = C. Essentially, it signifies the absence of bias in selection or mutation forces within complementary DNA strands [30].

**Figure 4:**
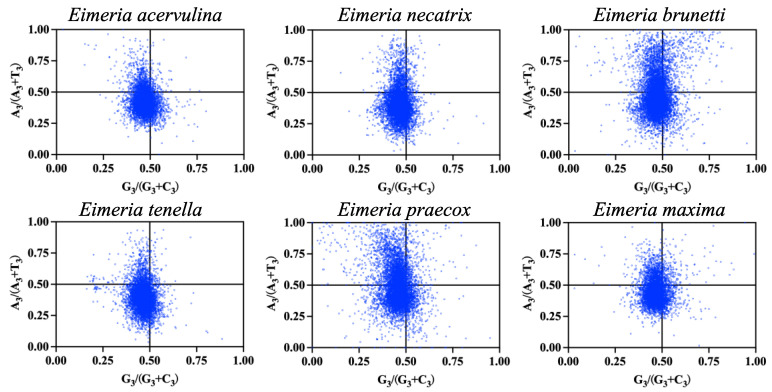
The PR2-plot analysis was performed on protein-coding genes across six *Eimeria* species. The species examined include *E. acervulina*; *E. necatrix*; *E. brunetti*; *E. tenella*; *E. praecox* and *E. maxima*.

The results indicate that the majority of genes were distributed in the third quadrant among the six *Eimeria* species. The mean values of GC bias [G3/(G3 + C3)] ranged from 45.41 (*E. praecox*) to 47.23 (*E. brunetti*), and AT bias [A3/(A3 + T3)] ranged from 39.62 (*E. tenella*) to 49.17 (*E. praecox*), suggesting a pronounced preference for C over G and T over A at the third codon position (Figure 4). This implies a tendency for pyrimidine over purine usage in the third base of codons within *Eimeria* genomes. Therefore, the CUB of coding genes in the six *Eimeria* species is influenced not only by mutations but also significantly by other factors such as natural selection.

### 2.5. Neutrality Plot Analysis

**Figure 5:**
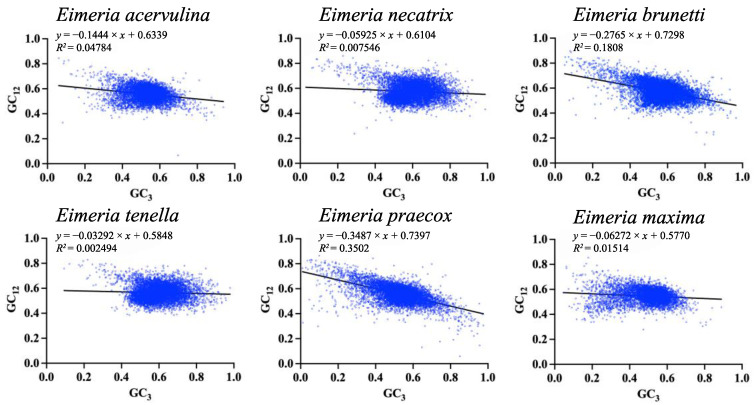
The neutrality plot analysis was conducted on GC12 and GC3 for the protein-coding genes across six *Eimeria* species. The species analysed are as follows: *E. acervulina*; *E. necatrix*; *E. brunetti*; *E. tenella*; *E. praecox* and *E. maxima*.

To provide additional insights into the impact of mutational pressure and natural selection of CUB on *Eimeria* genomes, we performed a neutrality plot analysis with GC12 and GC3 values in each gene. When nucleotide changes result in alterations to the encoded amino acid, it signifies the presence of selection pressure. Conversely, a correlation between GC12 and GC3 likely indicates the influence of mutational forces, as the force-shaping codon bias operates across all codon positions [31]. If the slope of the regression line approaches 1, suggesting that genes are distributed predominantly along the diagonal, it means that CUB is only influenced by mutational pressure alone. As the slope gradually decreases or even diminishes to 0, the impact of natural selection on CUB progressively strengthens. The results reveal statistically significant negative correlations between GC12 and GC3 across all six *Eimeria* species (*p* < 0.0001), with r values ranging from −0.04994 (*E. tenella*) to −0.5918 (*E. praecox*), and slope values of the regression line ranging from 0.03292 (*E. tenella*) to 0.3487 (*E. praecox*). The lower slope values indicate that mutational pressure is not the predominant pressure, which means that, in *E. praecox*, the proportion of neutrality (mutation pressure) was 34.87%, while the proportion of constraint on GC3 (natural selection) was 65.13%, contrasting with 3.292% and 96.708%, respectively, in *E. tenella* (Figure 5). The neutrality plot revealed that selection pressure exerted a greater influence than mutational pressure for CUB in six *Eimeria* genomes.

### 2.6. Correspondence Analysis

**Figure 6:**
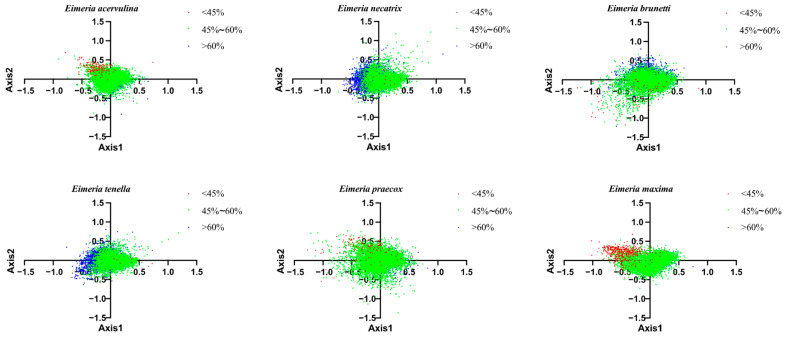
Correspondence analysis (COA) utilising the relative synonymous codon usage (RSCU) values obtained from protein-coding genes within six *Eimeria* species. In the graphical representation, red denotes genes with GC content falling below 45%, green represents genes with GC content ranging between 45% and 60%, and blue indicates genes with GC content exceeding 60%. The species included are labelled as follows: *E. acervulina*; *E. necatrix*; *E. brunetti*; *E. tenella*; *E. praecox* and *E. maxima*.

A correspondence analysis (COA) was conducted using the RSCU values of genome-wide protein-coding genes across six *Eimeria* species to examine codon biases. Axis 1, Axis 2, Axis 3, and Axis 4 accounted for 13.20%, 8.03%, 4.24%, and 3.75% of the average variation rates, respectively, with the first four axes collectively contributing to an average cumulative variation of 29.23%. Axis 1 emerged as the primary factor influencing CUB. Pearson correlation analysis demonstrated a significant relationship (*p* < 0.05) between the coordinate value of genes on the first axis and ENC, GC3s, GC3, and GC values across the six *Eimeria* species. To investigate the impact of GC content on CUB within these species, genes were colour-coded based on their GC content. The findings revealed a concentration of genes with GC content exceeding 60% or falling below 45% on the left or right side of the coordinate axis, whereas genes with GC content ranging between 45% and 60% were distributed on both sides of the axis (Figure 6). This observation underscores the influence of both selection pressure and gene mutation on CUB in *Eimeria* genomes.

### 2.7. Optimal Codon Analysis of Eimeria Genomes

**Figure 7:**
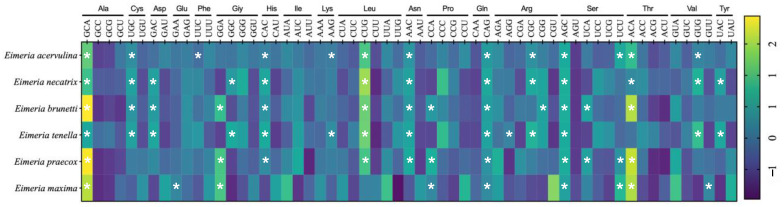
In the examination of optimal codon usage in six *Eimeria* species, codons meeting the criteria of ΔRSCU > 0.08 and RSCU > 1 are indicated in a single asterisk. Codons exhibiting high ΔRSCU are highlighted in yellow, while codons with low ΔRSCU are denoted in purple.

The comparative analysis revealed that *E. acervulina*, *E. necatrix*, *E. brunetti*, *E. tenella*, *E. praecox*, and *E. maxima* had 14, 13, 13, 15, 11, and 11 optimal codons (ΔRSCU > 0.08 and RSCU > 1), respectively. The majority of optimal codons in *Eimeria* species end with C or G, except for *E. praecox* and *E. maxima*. Among these, GCA, CAG, and AGC are the most commonly used optimal codons favoured in six *Eimeria* species. Following closely are CAC, CUG, AAC, and ACA, which are preferred in five *Eimeria* species. UGC is the top optimal codon in four *Eimeria* species. GAC, GGA, CCA, CGC, UCU, and GUG serve as optimal codons in three out of six *Eimeria* species. GGC, AAG, UCA, and UAC serve as optimal codons in two *Eimeria* species. Additionally, individual *Eimeria* species exclusively favour GAA, UUU, AGG, CGG, and GUU as their optimal codons (Figure 7, Table S2).

### 2.8. Comparative Analysis of Codon Usage between Eimeria and Other Organisms

Utilising the Codon Usage Database, we conducted a comparative analysis of codon usage frequencies between *E. tenella* and various other species to ascertain similarities in codon usage preferences. We specifically examined codons in *E. tenella* (ET) that displayed frequency ratios ≥2 or ≤0.5 when compared with those in *Gallus gallus* (GG), *Toxoplasma gondii* (TG), *Plasmodium vivax* (PV), *Cryptosporidium parvum* (CP), *Entamoeba histolytica* (EH), *Mus musculus* (MM), and *Homo sapiens* (HS). For each species pair, we identified 2, 5, 18, 35, 43, 4, and 3 such codons, respectively. A lower count of codons suggests a smaller disparity in synonymous CUB between the two species. Consequently, *E. tenella* displays closer alignment in codon usage preferences with *Gallus gallus*, *Toxoplasma gondii*, *Mus musculus*, and *Homo sapiens*, while notable discrepancies are observed with *Plasmodium vivax*, *Cryptosporidium parvum*, and *Entamoeba histolytica* (Table 2). The identical analysis performed on *E. acervulina*, *E. necatrix*, *E. brunetti*, *E. praecox*, and *E. maxima* produced findings analogous to those observed in *E. tenella*.

## 3. Discussion

Numerous studies have demonstrated that the CUB is influenced by a complex interplay between mutational processes and selective pressures throughout the evolutionary history of organisms [32]. Codon selection plays a crucial role in regulating gene expression, as optimal codons can enhance both the efficiency and accuracy of translation [33]. A plethora of biochemical, genetic, biophysical, and bioinformatics investigations have demonstrated that codon preference impacts various gene regulatory mechanisms, encompassing protein translation, co-translational folding, transcription, and post-transcriptional regulatory processes [34]. Moreover, codon usage profoundly influences gene expression and protein functionality across a spectrum of organisms, spanning from lower to higher organisms. In the context of expressing heterologous genes, optimising the codons of the target gene to match the preferred codons of the host species can significantly improve gene expression efficiency [35]. Several studies have highlighted the significant impact of base composition on codon preference [36]. Furthermore, the preferences for the utilisation of bases, codons, and amino acids are also influenced by factors such as gene expression levels, gene functions, and the evolutionary development of the species [37].

The current investigation focuses on elucidating CUB within the genomes of six *Eimeria* species. Remarkably, the genomes of *Eimeria* species are GC-rich, with these six species displaying a relatively elevated GC content in their genomes. Across all six genomes, the GC3 content ranges from 48.71% to 59.75%, surpassing that of GC2, with the distribution pattern being GC1 > GC3 > GC2. This investigation demonstrates an unbalanced utilisation of GC and AT bases at the third codon position in *Eimeria* genomes. In four-codon amino acids, G/C-ending codons appear to be preferred over A/T-ending codons. Additionally, codons ending in C are favoured over those ending in G, and codons ending in T are favoured over those ending in A, demonstrating a preference for pyrimidine bases in the third position. These observations suggest an influence of GC3 bias on codon usage patterns. The human genome exhibits a preference for G or C, particularly in synonymous codons terminating with C [38]. This preference for C or G in the third codon position is also observed in species such as *Caenorhabditis elegans*, *Daphnia pulex*, and *Drosophila melanogaster* [39]. Conversely, species like *Borrelia burgdorferi*, *Mycoplasma capricolum*, *Onchocerca volvulus*, and *Plasmodium falciparum* exhibit a preference for A or T in the third codon position [40,41,42]. Studies in mammals have revealed that genes with higher GC content tend to exhibit elevated expression levels compared to those with lower GC content [43], warranting further investigation into the potential correlation between *Eimeria* gene expression levels and GC content.

The CDSs in the six *Eimeria* species displayed an average ENC ranging from 47.37 ± 8.70 to 51.93 ± 5.65. If codon usage is solely influenced by GC3 content, it suggests the presence of mutational pressure. In such instances, the ENC values tend to be slightly higher than the expected ENC curve [44]. In all six *Eimeria* genomes, most CDSs showed ENC values below the expected curve, with from 1.22% to 7.32% of their genes exhibiting an ENC below 35, suggesting a significant preference for specific codons and highlighting the dominant influence of natural selection pressure. Following this, we have also explored the influence of natural selection pressure through the neutrality plot analysis. Mutational pressure is inferred as the primary determinant of CUB when the gradient of the regression line approaches 1 and the correlation between GC12 and GC3 achieves statistical significance. Conversely, gradients nearing 0 or displaying a nearly horizontal trajectory suggest that natural selection pressure predominantly shapes CUB. In the neutrality plot analysis, with the slope of the regression line ranging from 0.03292 to 0.3487 in six *Eimeria* species, most genes tended to diverge considerably from the slope of the regression line, further confirming the dominance of natural selection over mutational forces. Both the neutrality plot and PR2-plot analyses provided compelling evidence supporting the involvement of natural selection in codon bias within Eimeria. This study’s findings underscore that despite variations in codon usage indicators within *Eimeria* species, the CUB of protein-coding genes observed in these six *Eimeria* species is influenced by both natural selection pressures and mutational processes. Notably, all six *Eimeria* species experienced robust natural selection pressures on their protein-coding genes, particularly when considering the base composition. Moreover, no correlation was observed between ENC and GRAVY or AROMO, indicating no influence of hydrophobicity or aromaticity on CUB. Similarly, no significant associations were found between CAI, GRAVY, and AROMO, suggesting minimal impact of these factors on gene expression.

This study analysed the CUB of six *Eimeria* species at the whole-genome level. The results indicate that there are 26–31 preferred codons in these genomes, with 11–15 of them being optimal codons. The CUB of six *Eimeria* species are similar to those of *Gallus gallus*, *Toxoplasma gondii*, *Mus musculus*, and *Homo sapiens*, but markedly different from those of *Plasmodium vivax*, *Cryptosporidium parvum*, and *Entamoeba histolytica*. These findings suggest a potential co-evolutionary relationship between *Eimeria* and host genomes, all six *Eimeria* species are well adapted to *Gallus gallus*. Phylogenetic analysis reveals that *Eimeria* is more closely related to T. gondii, implying that species with closer phylogenetic relationships tend to share more similar CUB. Coccidiosis has emerged as a significant health threat in poultry, necessitating urgent efforts toward vaccine development and therapeutic discovery. *Eimeria* species displayed significant adaptation to sequences from both Mus musculus and Homo sapiens, as evidenced by CUB. Therefore, cell lines derived from bats and humans may provide robust support for *Eimeria* gene replication. Our findings provide valuable insights for selecting optimal experimental cell lines for vaccine development, heterologous gene expression studies, and research related to pathogenicity. Identifying distinct codon patterns is essential for understanding gene expression and evolutionary impacts on the genome. It also aids in phylogenetic analysis and optimising gene expression through codon optimisation. Numerous studies emphasise the association between CUB and gene expression levels, impacting translation efficiency throughout the proteome [45]. Translational selection affects both codon and amino acid usage, with highly expressed genes favouring amino acids with low or intermediate size/complexity scores, such as alanine and glycine, and disfavouring those with high scores, such as cysteine [46]. 

Different organisms use varying frequencies of different codons to encode the same amino acid. To enhance the expression of exogenous proteins, optimising inserted foreign gene sequences based on the codon bias of the target organism is essential. This optimisation primarily aims to reduce rare codon usage, thereby increasing transcription speed and lowering error rates [47]. Moreover, optimised genes often feature higher guanine and cytosine nucleotide content, which enhances mRNA stability and potentially improves mRNA transport efficiency from the nucleus to the cytoplasm, thus boosting exogenous protein expression. Advances in biotechnology have facilitated the prediction of optimised gene sequences based on the target protein’s sequence, followed by the synthesis of these optimised foreign genes using artificial methods. In vaccine development, codon optimisation has proven effective in enhancing antigen protein expression levels and is widely applied with successful outcomes [48]. Regarding the adaptation of *Eimeria* species to host species, they typically rely on the host cell’s gene expression machinery to synthesise their own proteins. This includes utilising the host’s tRNA molecules for translation. *Eimeria* species have evolved mechanisms to interact with and manipulate host cell processes, allowing them to exploit host resources for their own replication and survival within the host’s intestinal cells during infection [49]. This adaptation is critical for their lifecycle and pathogenicity in poultry. Future research should investigate correlations among codon usage, amino acid frequency, and expression levels. Furthermore, our findings offer theoretical guidance for the functional genomic study of *Eimeria* genomes and the vaccine strategy of coccidiosis in poultry.

## 4. Materials and Methods

### 4.1. Genomic Data

The genomic data and annotations for *E. acervulina*, *E. necatrix*, *E. brunetti*, *E. tenella*, *E. praecox*, and *E. maxima* were retrieved from the NCBI genome database (https://www.ncbi.nlm.nih.gov/genome/, accessed on 1 June 2024) (Table S1). A customised Python script was utilised to filter genes based on specific criteria pertaining to CDS: sequences exceeding 300 base pairs in length, with the number of bases being a multiple of three, and containing complete start and stop codons. Subsequently, a total of 5717 coding genes for *E. acervulina*, 7222 for *E. necatrix*, 8006 for *E. brunetti*, 6519 for *E. tenella*, 7096 for *E. praecox*, and 5205 for *E. maxima* were identified and retained in the filtered sequence files, respectively.

### 4.2. Calculation of Codon Related Parameters

Among common codons, ATG and TGG encode only one amino acid, while TAA, TAG, and TGA function as stop codons. These five codons were excluded, and subsequent bioinformatics analysis focused on the remaining codons. The effective number of codons (ENC) reflects the codon diversity within the gene. An ENC value of 20 suggests one codon per amino acid, while 61 indicates the average usage of each codon. The nucleotide composition of CDSs was assessed, focusing specifically on various GC-related metrics. GC signifies the total count of guanine (G) and cytosine (C) nucleotides in each gene, while GC1, GC2, and GC3 represent the counts of G and C nucleotides at the first, second, and third positions of each codon in the gene, respectively. Additionally, GC12 denotes the average count of G and C nucleotides at the first and second positions of codons. Furthermore, T3s, C3s, A3s, and G3s denote the frequencies of thymine (T), cytosine (C), adenine (A), and guanine (G) nucleotides, respectively, at the third position of codons within CDSs. Lastly, GC3s represent the GC content specifically at the third position of synonymous codons. General average hydropathicity (GRAVY) values, ranging from −2 to 2, are obtained by summing the hydropathy values of amino acids in polymerase gene sequences and multiplying by the number of residues. Positive and negative GRAVY values represent hydrophobic and hydrophilic proteins, respectively. The aromaticity (AROMO) value reflects the frequency of aromatic amino acids (Phe, Tyr, and Trp). GRAVY and AROMO values serve as indicators of amino acid usage, and changes in amino acid composition can impact codon usage analysis results. Relative synonymous codon usage (RSCU) denotes the ratio of the observed frequency of codons to the expected frequency, assuming equal usage of all synonymous codons for the same amino acids. RSCU values greater than 1 signify positive codon bias, values less than 1 indicate negative bias, and values equal to 1 denote random codon usage. DAMBE 7.3.11 software [50], CodonW 1.4.2 software [51], and custom Biopython scripts were utilised for analysing the aforementioned parameters.

### 4.3. ENC-Plot Analysis

The ENC-Plot is commonly utilised to assess whether codon usage in a particular gene is influenced solely by mutation or by other factors. The ENC-Plot was generated using R programming language, with ENC values plotted on the ordinate and GC3s values on the abscissa. The expected curve of ENC is calculated by the formula: ENC_exp_ = 2 + GC3s + 29/[GC3s^2^ + (1 − GC3s)^2^] [52]. When the data points cluster around this expected curve, it suggests that mutation pressure independently contributes to codon bias formation. Conversely, if data points deviate significantly from the expected curve, it indicates the involvement of other factors, such as natural selection, in shaping codon bias. Furthermore, we evaluated the discrepancies between the expected and actual ENC values using the ENC_ratio_ index by the formula: ENC_ratio_ = (ENC_exp_ − ENC_obs_)/ENC. The ENC_ratio_ value quantifies the extent of variation between the expected and observed ENC values.

### 4.4. PR2-Plot Analysis

The PR2-plot was generated using the R programming language to analyse the proportional relationship between purine and pyrimidine at the third base of each four-codon degenerate amino acid. The four-codon degenerate amino acids are alanine (GCT, GCG, GCC, GCA), glycine (GGT, GGG, GGC, GGA), proline (CCA, CCC, CCT, CCG), threonine (ACC, ACA, ACG, ACT), valine (GTT, GTG, GTC, GTA), leucine (CTA, CTC, CTG, CTT), serine (TCA, TCC, TCG, TCU), and arginine (CGA, CGC, CGG, CGU). We employed A3/(A3 + T3) as the vertical axis and G3/(G3 + C3) as the horizontal axis, where A3, T3, G3, and C3 denote the content of A, T, G, and C in the third codon position, respectively. These values for the four-codon degenerate amino acids were computed using a custom Biopython script. The vectors extending from the centre point to other points delineate the preferred orientation and strength of purine or pyrimidine bias on the third base of codons.

### 4.5. Neutrality Plot Analysis

The neutrality plot is primarily employed to examine the relationship between GC12 and GC3. In this study, the neutrality plot was constructed using the R programming language to assess the intricate interplay between mutational pressure and natural selection in shaping CUB within genes in six *Eimeria* species. GC3 values were plotted on the abscissa, while GC12 values were plotted on the ordinate. A regression line through the plotting of GC3s against GC12s was applied to fit the plot. The significance of the correlation observed in relation to the slope of the regression line indicates the impact of mutational forces on the overall outcome [53].

### 4.6. Analysis of Optimal Codons in Eimeria Genomes

The ENC values of CDS from six *Eimeria* species were computed, respectively. Subsequently, CDS sequences from each species were sorted based on their ENC values, with the lowest and highest 10% identified and separated to construct high- and low-expression libraries, respectively. Specifically, sequences with low ENC values were categorised as high-expression libraries, while those with high ENC values formed the low-expression libraries. Following this, the RSCU and ΔRSCU values were calculated for each group. Codons with RSCU values exceeding 1 were considered high-frequency codons, whereas those with ΔRSCU values exceeding 0.08 were deemed high-expression codons. Codons meeting both criteria were designated as optimal codons.

### 4.7. Comparative Analysis of Codon Usage between Eimeria and Other Organisms

The codon usage of the *Eimeria tenella* genome was compared with that of *Gallus gallus*, *Toxoplasma gondii*, *Plasmodium vivax*, *Cryptosporidium parvum*, *Entamoeba histolytica*, *Mus musculus*, and *Homo sapiens*. Codon usage tables for various species were obtained from the Codon Usage Database (http://www.kazusa.or.jp/codon/, accessed on 1 June 2024) [54]. A frequency ratio between 0.50 and 2.00 for synonymous codon usage in different species suggests a tendency for both species to employ the same synonymous codon. Conversely, if the ratio falls outside the 0.50–2.00 range, it indicates a preference for a specific synonymous codon in one or both of the compared species.

### 4.8. Statistical Analysis

The data are expressed as means ± standard deviation (SD), and statistical analyses were conducted using GraphPad Prism 8. Significance levels were set at * *p* < 0.05 and ** *p* < 0.001. Group mean differences were assessed through either one-way ANOVA or Student’s *t*-test.

## Figures and Tables

**Table 1:** Average GC content and ENC values of protein-coding genes in six *Eimeria* species.

Species	GC	GC1	GC2	GC3	GC3s	ENC
*E. acervuline*	54.77	62.71	48.83	52.78	51.64	51.92
*E. necatrix*	58.24	64.61	50.40	59.72	58.80	49.30
*E. brunetti*	57.12	66.09	48.45	56.83	55.93	47.67
*E. tenella*	57.59	63.64	49.38	59.75	58.78	50.25
*E. praecox*	54.59	65.60	45.84	52.33	51.37	47.37
*E. maxima*	52.67	61.99	47.30	48.71	47.43	51.17

GC: GC content of all codons; GC1: GC content at the first position of codons; GC2: GC content at the second position of codons; GC3: GC content at the third position of codons; GC3s: GC content at the third position of synonymous codons. ENC: effective number of codons.

**Table 2:** Comparison of synonymous codon usage between *E. tenella* and other species.

Amino Acid	Codon	Codon Frequency								Ratio						
		ET	GG	TG	PV	CP	EH	MM	HS	ET/GG	ET/TG	ET/PV	ET/CP	ET/EH	ET/MM	ET/HS
Phe	UUU	16.3	16.8	13.3	22.6	34.6	31.3	17.2	17.6	0.97	1.23	0.72	0.47	0.52	0.95	0.93
	UUC	17.3	20.2	25	17	12.1	11.3	21.8	20.3	0.86	0.69	1.02	1.43	1.53	0.79	0.85
Leu	UUA	6.2	7	2.6	13.7	33.7	43.6	6.7	7.7	0.89	2.38	0.45	0.18	0.14	0.93	0.81
	UUG	18.7	12.6	14.5	18.9	17.3	6.3	13.4	12.9	1.48	1.29	0.99	1.08	2.97	1.4	1.45
	CUU	16.4	12.4	15	9	20.3	26.9	13.4	13.2	1.32	1.09	1.82	0.81	0.61	1.22	1.24
	CUC	17.8	16.8	27.1	14	5.2	2.1	20.2	19.6	1.06	0.66	1.27	3.42	8.48	0.88	0.91
	CUA	8.2	6	3.8	10	10.4	2.7	8.1	7.2	1.37	2.16	0.82	0.79	3.04	1.01	1.14
	CUG	41.4	38.5	24	17.4	3.7	0.6	39.5	39.6	1.08	1.73	2.38	11.19	69	1.05	1.05
Ile	AUU	14.7	16.8	12.2	22.7	49.3	59.4	15.4	16	0.88	1.2	0.65	0.30	0.25	0.95	0.92
	AUC	11.4	22	17.4	16.9	10.6	5.9	22.5	20.8	0.52	0.66	0.67	1.08	1.93	0.51	0.55
	AUA	7.9	8.8	2.9	16.5	24.4	14.6	7.4	7.5	0.90	2.72	0.48	0.32	0.54	1.07	1.05
Val	GUU	15.3	13.1	14.7	12.8	24.4	42	10.7	11	1.17	1.04	1.2	0.63	0.36	1.43	1.39
	GUC	14.1	13.6	25.4	10	4.8	4.3	15.4	14.5	1.04	0.56	1.41	2.94	3.28	0.92	0.97
	GUA	7.5	7.8	5.7	15	15.8	16.9	7.4	7.1	0.96	1.32	0.5	0.47	0.44	1.01	1.06
	GUG	19.9	28.2	21.4	19.7	4.9	2.6	28.4	28.1	0.71	0.93	1.01	4.06	7.65	0.7	0.71
Ser	UCU	14.8	14.1	20.3	9.1	28.3	17.1	16.2	15.2	1.05	0.73	1.63	0.52	0.87	0.91	0.97
	UCC	12	15.7	15.2	13.7	7.9	1.5	18.1	17.7	0.76	0.79	0.88	1.52	8	0.66	0.68
	UCA	11	11.6	8.1	9.2	31.4	27.8	11.8	12.2	0.95	1.36	1.2	0.35	0.4	0.93	0.9
	UCG	9.8	5.2	19	8.2	4.3	0.7	4.2	4.4	1.88	0.52	1.2	2.28	14	2.33	2.23
	AGU	8.7	11.2	9.3	12.7	18.4	17	12.7	12.1	0.78	0.94	0.69	0.47	0.51	0.69	0.72
	AGC	28.6	20.2	16.1	17.8	8.6	1.6	19.7	19.5	1.42	1.78	1.61	3.33	17.88	1.45	1.47
Pro	CCU	14.2	15.3	15.4	5.7	12.9	8.8	18.4	17.5	0.93	0.92	2.49	1.10	1.61	0.77	0.81
	CCC	14.4	17	13.6	9.4	2.8	0.7	18.2	19.8	0.85	1.06	1.53	5.14	20.57	0.79	0.73
	CCA	13.1	15.7	10.4	13.4	20.8	27.4	17.3	16.9	0.83	1.26	0.98	0.63	0.48	0.76	0.78
	CCG	11.1	7.8	17.3	4.1	1.9	0.2	6.2	6.9	1.42	0.64	2.71	5.84	55.5	1.79	1.61
Thr	ACU	13.6	13.3	12.3	11.8	21.6	25.5	13.7	13.1	1.02	1.11	1.15	0.63	0.53	0.99	1.04
	ACC	10.2	16.5	13	15.4	5.5	2.9	19	18.9	0.62	0.78	0.66	1.85	3.52	0.54	0.54
	ACA	15.6	16.1	13.2	14.3	20.4	27.5	16	15.1	0.97	1.18	1.09	0.76	0.57	0.98	1.03
	ACG	11.5	7.7	16.4	11.1	2.6	1.1	5.6	6.1	1.49	0.7	1.04	4.42	10.45	2.05	1.89
Ala	GCU	34.6	20.8	20.5	11.7	17	26.9	20	18.4	1.66	1.69	2.96	2.04	1.29	1.73	1.88
	GCC	20.5	22.9	22.6	16.3	3.9	2.7	26	27.7	0.90	0.91	1.26	5.26	7.59	0.79	0.74
	GCA	42.5	19	20.8	26.6	17.5	27.5	15.8	15.8	2.24	2.04	1.6	2.43	1.55	2.69	2.69
	GCG	19.9	9.1	31.5	13.3	1.9	0.5	6.4	7.4	2.19	0.63	1.5	10.47	39.8	3.11	2.69
Tyr	UAU	7.5	11.8	5.1	15.1	25.9	32.1	12.2	12.2	0.64	1.47	0.5	0.29	0.23	0.61	0.61
	UAC	12.3	17.8	14.5	26.8	8.9	3.8	16.1	15.3	0.69	0.85	0.46	1.38	3.24	0.76	0.8
His	CAU	8.6	9.5	7.9	6.9	14.1	15.8	10.6	10.9	0.91	1.09	1.25	0.61	0.54	0.81	0.79
	CAC	13.1	14.4	13.4	10.2	4.6	2.1	15.3	15.1	0.91	0.98	1.28	2.85	6.24	0.86	0.87
Gln	CAA	18.5	12.1	13.7	20.6	27.5	37	12	12.3	1.53	1.35	0.9	0.67	0.5	1.54	1.5
	CAG	44.7	32.6	24.5	14.8	9.1	1.7	34.1	34.2	1.37	1.82	3.02	4.91	26.29	1.31	1.31
Asn	AAU	10.2	16.9	10.1	34.8	58	48.1	15.6	17	0.60	1.01	0.29	0.18	0.21	0.65	0.6
	AAC	17.6	22.5	18.9	32.7	16.8	8	20.3	19.1	0.78	0.93	0.54	1.05	2.2	0.87	0.92
Lys	AAA	21.7	27.3	18.4	53.7	48.5	64.1	21.9	24.4	0.79	1.18	0.4	0.45	0.34	0.99	0.89
	AAG	24	34.3	29.8	51.3	25.9	16.9	33.6	31.9	0.70	0.81	0.47	0.93	1.42	0.71	0.75
Asp	GAU	14.2	25.3	16.3	28.6	40.8	45	21	21.8	0.56	0.87	0.5	0.35	0.32	0.68	0.65
	GAC	24.2	24.9	34.3	26.2	10.7	8.4	26	25.1	0.97	0.71	0.92	2.26	2.88	0.93	0.96
Glu	GAA	28.4	31	32.7	54.1	50.4	65.5	27	29	0.92	0.87	0.52	0.56	0.43	1.05	0.98
	GAG	31.4	40.9	40.6	34.4	19.3	6.4	39.4	39.6	0.77	0.77	0.91	1.63	4.91	0.8	0.79
Cys	UGU	7.8	8.8	7.2	9.9	13.1	19.1	11.4	10.6	0.89	1.08	0.79	0.60	0.41	0.68	0.74
	UGC	19.2	13.3	12.4	10.5	6.5	2	12.3	12.6	1.44	1.55	1.83	2.95	9.6	1.56	1.52
Arg	CGU	8	5.4	8.2	1.5	3.6	4.4	4.7	4.5	1.48	0.98	5.33	2.22	1.82	1.7	1.78
	CGC	14.7	10.4	17.5	2.7	0.9	0.1	9.4	10.4	1.41	0.84	5.44	16.33	147	1.56	1.41
	CGA	6.3	5.3	12.7	2.3	2.1	3	6.6	6.2	1.19	0.5	2.74	3.00	2.1	0.95	1.02
	CGG	9.9	9.7	10.1	1.4	0.4	0.2	10.2	11.4	1.02	0.98	7.07	24.75	49.5	0.97	0.87
	AGA	10.4	12.2	12.8	10.9	25.7	26.3	12.1	12.2	0.85	0.81	0.95	0.40	0.4	0.86	0.85
	AGG	10.4	11.7	8.7	8.6	6.9	1.6	12.2	12	0.89	1.2	1.21	1.51	6.5	0.85	0.87
Gly	GGU	10.5	11.4	14.6	10.9	15.9	17.2	11.4	10.8	0.92	0.72	0.96	0.66	0.61	0.92	0.97
	GGC	24.1	19.7	28.5	12.4	5.4	0.6	21.2	22.2	1.22	0.85	1.94	4.46	40.17	1.14	1.09
	GGA	16.8	17.6	21.4	22.4	24.8	41.7	16.8	16.5	0.95	0.79	0.75	0.68	0.4	1	1.02
	GGG	15.6	16	14.6	10.6	5.7	2.6	15.2	16.5	0.98	1.07	1.47	2.74	6	1.03	0.95

ET: *E. tenella*; GG: *Gallus gallus*; TG: *Toxoplasma gondii*; PV: *Plasmodium vivax*; CP: *Cryptosporidium parvum*; EH: *Entamoeba histolytica*; MM: *Mus muscculus*; HS: *Homo sapiens*.

## Data Availability

Data is contained within the article and Supplementary Materials.

## Supplementary Materials

The supporting information can be downloaded at: https://www.mdpi.com/article/10.3390/ijms25158398/s1.
